# Physiological demands in simulated tennis matches and hitting tests take account of the translational and rotational kinetic energy ratio of the ball

**DOI:** 10.3389/fspor.2023.1113717

**Published:** 2023-02-13

**Authors:** Munenori Murata, Takashi Naito

**Affiliations:** ^1^ Department of Faculty of Sports and Life Science, National Institute of Fitness and Sports in Kanoya, Kanoya, Japan; ^2^ Faculty of Law, Hokkai-Gakuen University, Sapporo, Japan

**Keywords:** physiological demands, simulated match-play tennis, mechanical energy, forehand groundstroke, fatigue, blood lactate

## Abstract

Assessment of fatigue effect on hitting ability in tennis has been controversial in previous studies. The purpose of this study was to determine the relationship between player fatigue and groundstroke type in tennis. We hypothesized that subjects with higher blood lactate concentration during play would apply heavier spin to the ball. We divided players into two groups based on their blood lactate concentration during a pre-measured hitting test (HIGH and LOW). Each group performed a simulated match-play protocol consisting of repeated running and hitting tests, which simulated a three-set match. Heart rate, percent of heart rate reserve, oxygen uptake, pulmonary ventilation, and respiratory exchange were measured. The distance between the ball's landing point and the target, and the ball's kinematics, were recorded during the hitting test between sets. We found no significant difference in ball kinetic energy between groups, but the HIGH group hit the ball with a greater ratio of rotational kinetic energy to total kinetic energy. However, the progression of the simulation protocol did not affect physiological responses (including blood lactate concentration) or hitting ability. Therefore, it is suggested that the type of groundstrokes used by players is one of the factors that should be considered when discussing fatigue in tennis.

## Introduction

Tennis is a world-class competitive and recreational sport attracting millions of players and fans worldwide (1). Tennis has been the subject of many physiology, biomechanics, and game analysis studies, both in the laboratory and in actual competition situations. In laboratory studies, treadmill-based simulated match-play tennis protocols and ball feeder-based hitting tests are often employed. Such studies tend to focus on physiological responses related to fatigue (2, 3) and tennis-specific performance such as stroke velocity or accuracy (4, 5). It is recognized that tennis performance substantially depends on a complex interaction of physical fitness (aerobic and anaerobic) and tennis-specific skills (6). However, these factors have been considered separately in most previous studies; the simulated match-play tennis protocol did not include hitting motions (2, 3), and a hitting test did not evaluate increase in fatigue as the game progressed (4). In an actual match, tennis is characterized by explosive actions, including sprints and hitting motions such as service or stroke, and players' physiological responses constantly change throughout the match (7). We concluded that for examining fatigue during both the sprint and hitting phases, it would be more appropriate to combine both simulated match-play tennis protocol (contributing the sprint phase) and hitting test.

Previous studies concluded that intensive rallies mainly demand energy provision for bouts of high-intensity work *via* intramuscular phosphates and glycolysis (8). Lack of anaerobic capability in tennis players causes early fatigue, leading in turn to impairment of ground stroke accuracy (9). Fatigue in tennis has been investigated using measurements of blood lactate concentration (BLa) and discussed in review articles (1, 10). These authors had mentioned that the BLa during tennis play had indicated varying results in the literature (11, 12), and no consensus has been reached. These inconsistent results suggest that BLa is associated with various factors, including match time, individual techniques, play-style and emotional stress. When focusing on the hitting technique in an actual game, players hit the ball in various forms and often use the drive and flat shots in groundstroke. However, the effect of these shots on BLa has not been reported.

On the other hand, the mechanical energy required for each shot will depend on the energy acquired by the racket as a result of body movements and the efficiency of energy transfer between the racket and the ball. Cross and Lindsey (13) propose a method to estimate the speed and spin of the ball after impact with the racket. Calculating the mechanical energy of the ball based on this method (assuming the ball speed and spin to be zero before impact) indicates that the ball's kinetic energy decreases more when the ball impacts the string bed tangentially than under a normal impact. This can also be observed from the motion data of the ball and racket when players hit serves. Based on the ball speed and spin rate reported in a previous study (14), the estimated kinetic energy of the ball in the flat, slice, and kick serves of male professional players was 79.2 J, 66.0 J, and 56.1 J, respectively [the ball's mass and moment of inertia assumed to be 57.7 g and the $0.55\;\;mr^{2}\;\;\mathrm{kg}\cdot m^{2}$ (15), where *m* and *r* are the ball's mass and radius]. However, no difference in the racket speed at impact was reported for these three types of serves (16). In groundstrokes, players hit the ball at various angles of impact (17), and the energy required for each shot may differ depending on whether the player applies greater or lesser spin to the ball. Since more rotation elicits a higher physical strain, it is thus possible that there might be a trend in the number of rotations when grouping by high and low values of BLa.

The mean duration of work periods during a match has been reported as approximately 4–7 s (18) and that of rest periods as 10–20 s (1). Average oxygen uptake values were above 80% maximum oxygen uptake (VO_2_max) during intensive rallies, but at approximately 50–60% VO_2_max (23–29 mL/kg/min) during match-play tennis including both sprint and hitting motions (1, 4), which is in the moderate intensity range. We speculated that the simulated match-play tennis protocol without hitting motion results in a lower exercise intensity than that found in previous studies. It is thus possible that the lesser physical strain from only running may not elicit any difference in physiological responses even if grouped by BLa values, due to alternate replenishing of energy sources and restoration of homeostasis (by oxidative metabolism) in the intervals (19).

Therefore, the purpose of this study was to compare the effect of high and low BLa groups on (1) the kinetic energy of the ball during a hitting test and (2) physiological responses during a simulated match-play tennis protocol. It was hypothesized that the high BLa group would show higher rotational kinetic energy in the hitting test compared to the low BLa group. We also hypothesized that running simulations would show no difference in physiological responses between groups.

## Methods

### Participants

Fourteen tennis players were recruited [ten males; age: 21 ± 1 year, height: 1.72 ± 0.06 m, body mass (BM): 63.6 ± 5.4 kg, and four females; age: 19 ± 1 year, height: 1.69 ± 0.04 m, BM: 62.2 ± 3.8 kg]. Participants were classified into two groups of seven participants each based on the median BLa in the first hitting test, HIGH (age: 20 ± 1 year, height: 1.73 ± 0.07 m, BM: 63.6 ± 6.8 kg, BLa: 2.58 ± 0.38 mmol/L) and LOW (age: 19 ± 1 year, height: 1.70 ± 0.03 m, BM: 62.2 ± 3.2 kg, BLa: 1.78 ± 0.25 mmol/L). Two women were included in each group (HIGH: 3.08 ± 0.11 mmol/L, LOW: 1.93 ± 0.25 mmol/L). All participants belonged to a national-level college tennis club, and their weekly training volume was approximately 15 h/week. One participant was a left-handed tennis player. The study protocol was approved by the National Institute of Fitness and Sports in Kanoya Ethics Committee (Permission number: 11–58), and participants provided their informed consent to participate prior to commencing the study. The study complied with the latest version of the Declaration of Helsinki, and was conducted according to international standards.

### Procedures

On arrival at an indoor hard court, players performed approximately 15 min of warm-up by running and hitting groundstrokes fed by a ball machine. After the warm-up, a finger prick BLa (Lactate Pro 2, Arkray Global Business Inc, Japan) measurement was taken. This measurement was also taken both immediately before and after the hitting test. Players were equipped with a portable metabolic system, which allowed the measurement of oxygen uptake (VO_2_), pulmonary ventilation (VE) and respiratory exchange ratio (RER) (MetaMax-3B, Cortex, Germany) and heart rate (HR) (RS-800, Polar, United States). Once the equipment was attached, players performed the hitting test and simulated match-play tennis. The total duration of the simulated match-play tennis was 6,108 s (1 h 41 min 48 s).

### Simulated match-play tennis protocol

**Figure 1:**
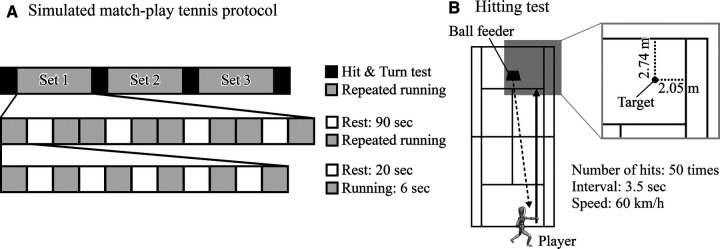
Overview of simulated match-play tennis protocol and hitting test. (**A**) Time structure of exercise and rest in the simulated match-play tennis protocol. From top to bottom, the structure of a match (3 sets), a set (8 games), and a game (5 points) are shown. The hitting test is shown in black, running in gray, and rest in white. (**B**) Overview of the hitting test. The target was set at 2.74 m from the baseline and 2.05 m from the singles line on the advantage side.

The simulated match-play tennis protocol employed was a modified running protocol designed by Lynch et al. (2). It was designed to simulate the temporal profile and volume of metabolic heat production of a professional tennis match, including the exact timing duration of rest breaks mandated by the Rules of Tennis. The protocol consisted of a series of 16 km/h sprints lasting an average of 6 s. To this end, players were asked to run 26.6 m in 6 s (16 km/h). This was followed by periods of recovery of 20 s. One 26 s cycle of exercise and recovery was recognized as one simulated “point”. Each simulated “game” consisted of six “points”, and each simulated “set” consisted of eight “games”. Each session comprised three simulated “sets”. Each mandated break of 90 s between odd games was implemented in accordance with International Tennis Federation rules (Figure 1A).

### Hitting test

The hitting test was performed before and after the simulated match-play tennis protocol and between sets. This test aimed to have the player perform groundstrokes with the same intensity as normally employed in an actual match. It consisted of returning a forehand down-the-line groundstroke to the opposite end of the court in a standing position behind the ball's bounce point (Figure 1B). A ball feeder (TQ-2000H II, Tanaka Electric Co., Japan) fed 50 balls at a frequency of one ball every 3.5 s, with a velocity of 60 km/h, 85 cm over the net, and landing 60 cm from the opposite baseline on the deuce side, in front of the player. Players were instructed to hit the balls at a submaximal velocity, returning the balls toward standard square landing zones (2.05 × 5.49 m) at the opposite end of the court. The ball trajectory was filmed by a digital video camera (FASTCAM Mini UX100, Photron Ltd., Japan). At the same time, the speed and spin rate of the ball just after hitting were measured by a laser Doppler ball kinematics analyzer (TrackMan tennis radar, TrackMan, Denmark). For left-handed players, the same hitting test was conducted on the opposite (left) side.

The two-dimensional coordinates of the ball landing points in digitized space were obtained from the video images using a custom program and converted to horizontal position in real space by the two-dimensional direct linear transformation method (20). Mean error of the landing point was defined as a relative position from the center of the target zone. The first and last 10 data points of each hitting test were excluded from the analysis. The kinetic energy of the ball was calculated by the following equations:

$$E_{t}=\frac{1}{2}mv^{2}$$

$$E_{r}=\frac{1}{2}\;I\omega^{2}$$

where $E_{t}$ is translational kinetic energy, *m* is the ball's mass, and *v* is the ball's speed; and $E_{r}$ is rotational kinetic energy, *I* is the moment of inertia, and *ω* is the angular speed. Total kinetic energy $\left(E_{k}\right)$ was then calculated as the sum of $E_{t}$ and $E_{r}$, and the ratio of rotational kinetic energy to total kinetic energy $\left(\text{\%}E_{r}=E_{r}/E_{k}\right)$ was also obtained.

### Calculation and statistical analysis

The HR, percentage of HR reserve (%HRR), VO_2_, VE and RER were averaged over the last min of the first and last game (i.e., games 1 and 8) in each set, and the hitting test. %HRR was calculated using the Karvonen formula: %HRR = (HR – rest HR)/(maximum HR – rest HR) × 100. Since maximum HR was not measured in the present study, a predicted value based on previously published results (220 - age) was adopted (21). All statistical computations were performed using the IBM SPSS Statistics 28 software package (SPSS Inc., USA). The distribution of the data was analyzed by a Shapiro–Wilk test, and Mauchly's test was used to examine sphericity. Physiological measurements except blood lactate concentration were analyzed by a three-way repeated measures analysis of variance (ANOVA) to compare data from different experimental groups, using group (2 levels: HIGH, LOW), set (3 levels: set 1, 2, 3) and time point in the set (3 levels: game 1, game 8, hitting test) as independent variables. The same ANOVA was performed for blood lactate concentration (2 levels: HIGH, LOW), set (4 levels: initial test before starting the simulated match-play tennis protocol and set 1, 2, 3) and timing of measurement (pre and post hitting test). The variables of the hitting test were similarly compared by two-way repeated measures ANOVA using group (2 levels: HIGH, LOW) and set as the independent variables. In the analysis of repeated measures, a Greenhouse–Geisser correction was used in the case of violations of the sphericity assumption. When a significant main effect or interaction effect was identified, a Holm's step-down procedure was performed for pairwise comparisons. The level of significance was set at *p* < 0.05. Cohen's d (*d*) was used as a measure of effect size for paired samples, with 0.2 to <0.6, ≥0.6 to <1.2, ≥1.2 to <2.0, ≥2.0 to <4.0, and ≥4.0 representing small, moderate, large, very large and extremely large treatment effects, respectively (22).

## Results

### Physiological measurements

**Figure 2:**
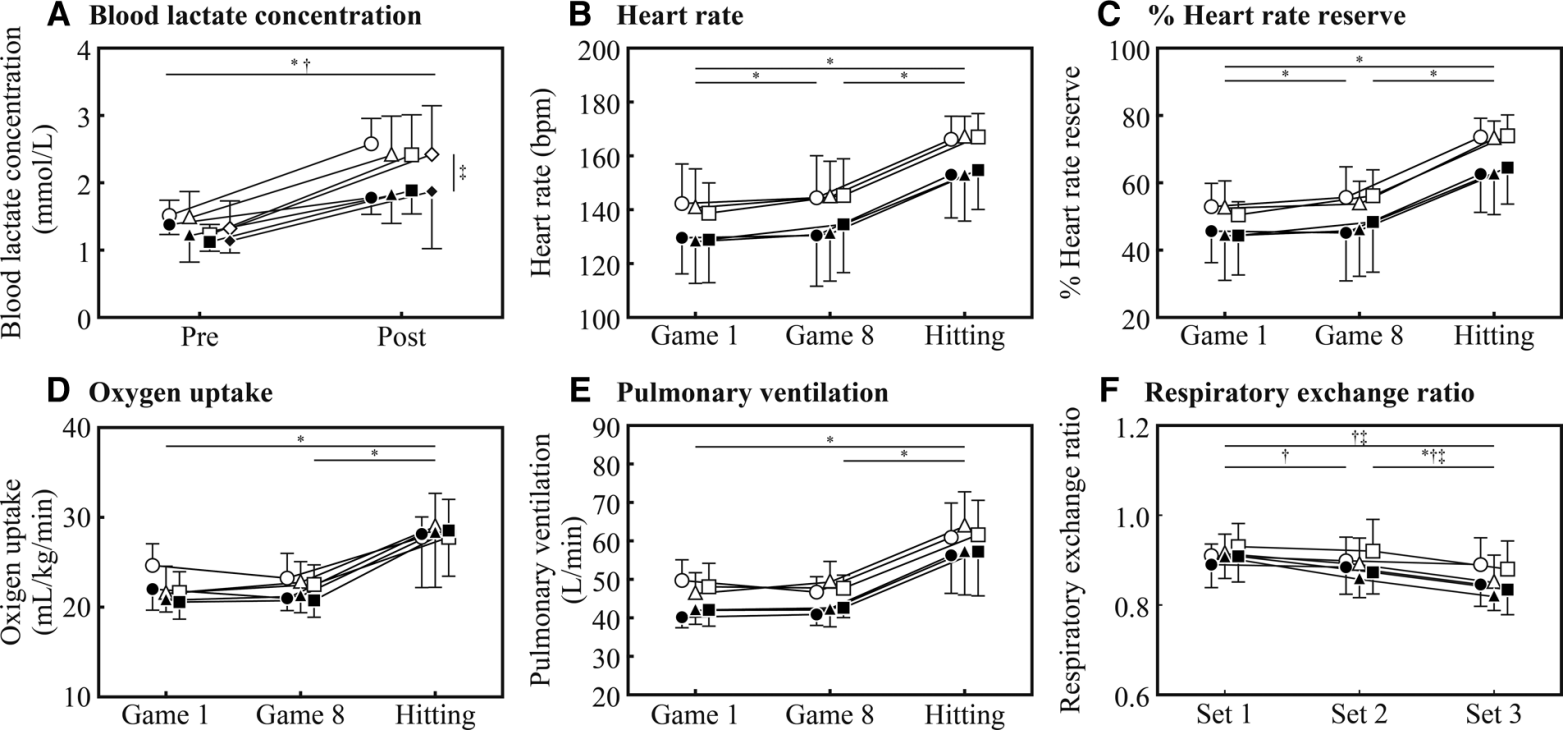
Physiological measurements at each time point. The HIGH group is indicated by a white symbol and the LOW group by a black symbol. (**A**) Blood lactate concentration pre and post hitting test before the simulated match-play tennis protocol and each set. Circles indicate pre-test, triangles, squares, and diamonds indicate 1st to 3rd sets, respectively. Significant differences between pre and post in HIGH and LOW are marked with “*” or “†”, respectively. Significant differences between HIGH and LOW in post are marked with “‡”. (**B–E**) Heart rate, % Heart rate reserve, oxygen uptake and pulmonary ventilation at each time point (game 1, game 8 and Hitting test). Circles, squares, and diamonds indicate 1st to 3rd sets, respectively. Significant differences between time points are marked with “*”. (**F**) Respiratory exchange ratio at each time point (set 1, set 2 and set 3). The HIGH group is indicated by a white symbol and the LOW group by a black symbol. Circles indicate pre-test, triangles, squares, and diamonds indicate game 1, game 8 and the hitting test, respectively. Significant differences in RER between sets at each time point, game 1, game 8 and the hitting test are marked with “*”, “†” and “‡”, respectively.

The analysis of blood lactate concentration showed a main effect of group [*F*(1, 12) = 6.281; *p* = 0.028; $\eta_{p}^{2}$ = 0.344] and pre/post [*F*(1, 12) = 69.409; *p* < 0.001; $\eta_{p}^{2}$ = 0.853] and an interaction between group and pre/post [*F*(1, 12) = 4.778; *p* = 0.049; $\eta_{p}^{2}$ = 0.285] (Table 1). Confirming the results of the post test (Figure 2A), BLa was higher in post than pre in both HIGH (*t* = 7.437; *p* < 0.001; *d* = 2.446; very large effect) and LOW (*t* = 4.345; *p* = 0.004; *d* = 1.429), and HIGH was higher than LOW in post (*t* = 3.291; *p* = 0.011; *d* = 1.413; large effect).

**Table 1:** Main effects and interactions of physiological measures.

		Main effect			Interaction			
		Group	Set	Game/Pre Post	Group × Set	Group × Game/Pre Post	Set × Game/Pre Post	Group × Set × Game/Pre Post
Blood lactate concentration	ndf	1	3	1	3	1	3	3
	ddf	12	36	12	36	12	36	36
	F	6.281	1.102	69.409	0.279	4.778	1.362	0.564
	*p*	0.028	0.361	<0.001	0.840	0.049	0.270	0.642
	$\eta_{p}^{2}$	0.344	0.084	0.853	0.023	0.285	0.102	0.045
Heart Rate	ndf	1	2	1.121	2	1.121	4	4
	ddf	12	24	13.455	24	13.455	48	48
	F	3.230	0.463	58.046	1.241	0.048	2.158	0.158
	*p*	0.097	0.635	<0.001	0.307	0.856	0.088	0.959
	$\eta_{p}^{2}$	0.212	0.037	0.829	0.094	0.004	0.152	0.013
% Hear rate reserve	ndf	1	2	1.142	2	1.142	4	4
	ddf	12	24	13.706	24	13.706	48	48
	F	3.406	0.877	64.515	0.643	0.285	2.068	0.414
	*p*	0.090	0.429	<0.001	0.535	0.632	0.100	0.798
	$\eta_{p}^{2}$	0.221	0.068	0.843	0.051	0.023	0.147	0.033
Oxygen uptake	ndf	1	2	1.078	2	1.078	4	4
	ddf	12	24	12.941	24	12.941	48	48
	F	0.592	4.433	42.644	1.052	0.889	5.815	1.415
	*p*	0.457	0.023	<0.001	0.365	0.371	<0.001	0.243
	$\eta_{p}^{2}$	0.047	0.270	0.780	0.081	0.069	0.326	0.105
Pulmonary ventilation	ndf	1	2	1.070	2	1.070	4	4
	ddf	12	24	12.845	24	12.845	48	48
	F	5.152	1.007	42.773	0.561	0.069	1.341	2.145
	*p*	0.042	0.380	<0.001	0.578	0.814	0.269	0.090
	$\eta_{p}^{2}$	0.300	0.077	0.781	0.045	0.006	0.100	0.152
Respiratory exchange ratio	ndf	12	2	2	2	2	4	4
	ddf	1	24	24	24	24	48	48
	F	1.466	30.182	3.115	1.339	0.478	4.870	1.015
	*p*	0.249	<0.001	0.063	0.281	0.626	0.002	0.409
	$\eta_{p}^{2}$	0.109	0.716	0.206	0.100	0.038	0.289	0.078

The factors are group, set, and game, but only lactate is considered as a Pre/Post instead of Game. Significant differences are highlighted. The ndf and ddf are numerator and denominator degrees of freedom, respectively.

The main effects of HR and %HRR were detected for game (HR: *F*(1.121, 13.455) = 58.046; *p* < 0.001; $\eta_{p}^{2}$ = 0.829, %HRR: *F*(1.142, 13.706) = 64.515; *p* < 0.001; $\eta_{p}^{2}$ = 0.843), but not for group (HR: *F*(1, 12) = 3.230; *p* = 0.097; $\eta_{p}^{2}$ = 0.212, %HRR: *F*(1, 12) = 3.406; *p* = 0.090; $\eta_{p}^{2}$ = 0.221) or set (HR: *F*(2, 24) = 0.463; *p* = 0.635; $\eta_{p}^{2}$^ ^= 0.037, %HRR: *F*(2, 24) = 0.877; *p* = 0.429; $\eta_{p}^{2}$^ ^= 0.068), and no interaction effects were observed (Table 1). In addition, HR (game 1 < hitting: *t* = 8.811; *p* < 0.001; *d* = 1.749; large effect, game 8 < hitting: *t* = 7.233; *p* < 0.001; *d* = 1.494; large effect) and %HRR (game 1 < hitting: *t* = 9.646; *p* < 0.001; *d* = 1.996; large effect, game 8 < hitting: *t* = 7.443; *p* < 0.001; *d* = 1.751; large effect) were significantly higher in the hitting test than in the simulated match-play tennis protocol (Figures 2B,C).

The main effects of VO_2_ and VE were detected for game (VO_2_: *F*(1.078, 12.941) = 42.644; *p* < 0.001; $\eta_{p}^{2}$ = 0.780, VE: *F*(1.070, 12.845) = 42.773; *p* < 0.001; $\eta_{p}^{2}$ = 0.781) but not for group in VO_2_ (VO_2_: *F*(1, 12) = 0.592; *p* = 0.457; $\eta_{p}^{2}$ = 0.047, VE: *F*(1, 12) = 5.152; *p* = 0.042; $\eta_{p}^{2}$ = 0.300). Moreover, VO_2_ showed an interaction effect between set and game [*F*(4, 48) = 5.815; *p* < 0.001; $\eta_{p}^{2}$ = 0.326] (Table 1). The hitting test was higher than in both VO_2_ (game 1 < hitting: *t* = 7.033; *p* < 0.001; *d* = 1.952; large effect, game 8 < hitting: *t* = 6.326; *p* < 0.001; *d* = 1.936; large effect) and VE (game 1 < hitting: *t* = 6.923; *p* < 0.001; *d* = 2.190; very large effect, game 8 < hitting: *t* = 6.813; *p* < 0.001; *d* = 2.166; very large effect) than the simulated match-play tennis protocol (Figures 2D,E). RER showed a main effect for set [*F*(2, 24) = 30.182; *p* < 0.001; $\eta_{p}^{2}$ = 0.716], and an interaction between set and game was also observed [*F*(4, 48) = 4.870; *p* = 0.002; $\eta_{p}^{2}$ = 0.289] (Table 1). The RER measured in game 8 decreased as the set progressed (set 1 > set 2: *t* = 3.857; *p* = 0.008; *d* = 0.692; moderate effect, set 1 > set 3: *t* = 8.016; *p* < 0.001; *d* = 1.438; large effect, set 2 > set 3: *t* = 4.159; *p* = 0.003; *d* = 0.746; moderate effect), and similarly the RER measured in the hitting test was lower in set 3 than in the other sets (set 1 > set 3: *t* = 6.579; *p* < 0.001; *d* = 1.181; moderate effect, set 2 > set 3: *t* = 4.159; *p* = 0.003; *d* = 0.746; moderate effect) (Figure 2F).

### Mechanical measurements

**Figure 3:**
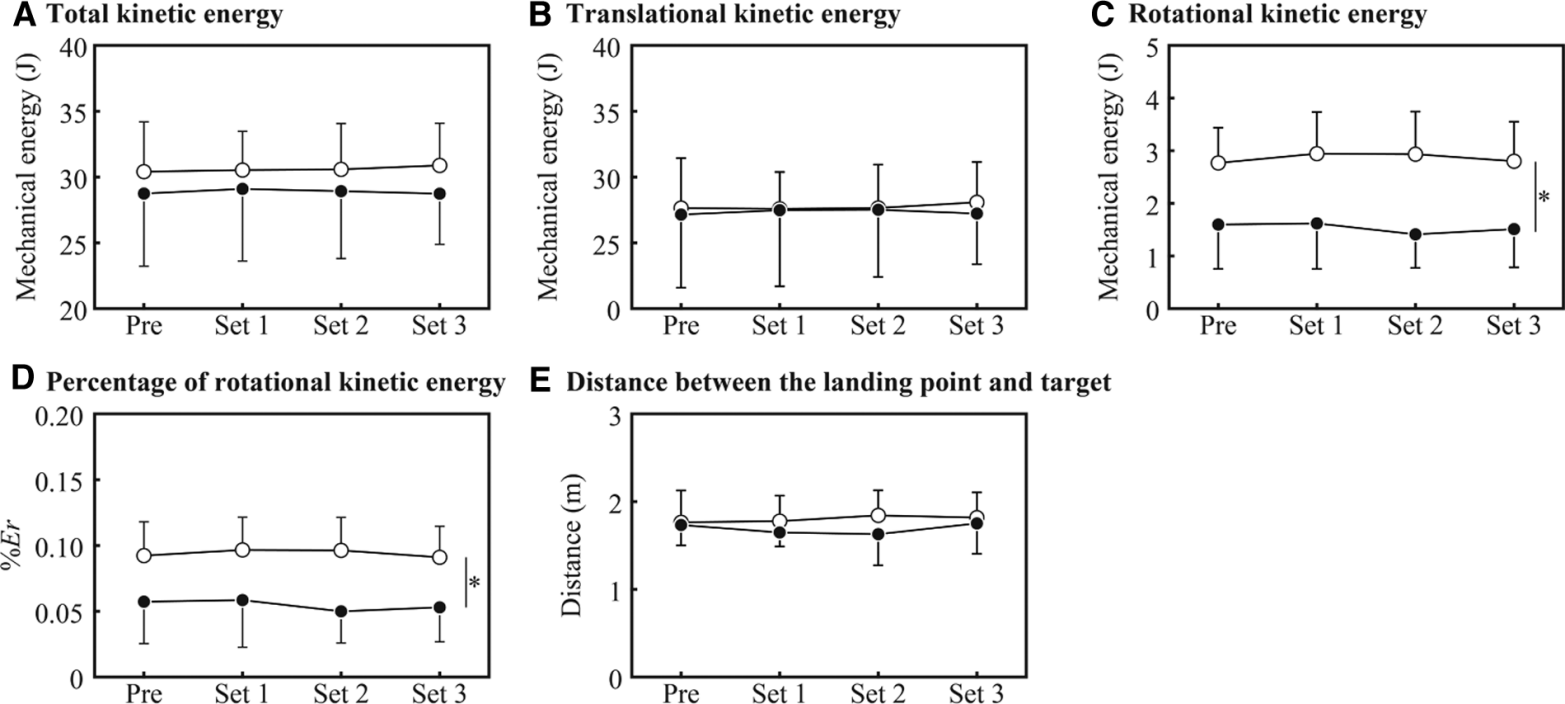
Results of the hitting test at each time point. White and black circles indicate the HIGH and LOW groups, respectively. An asterisk indicates a main effect between groups.

There was no main effect of group [*F*(1, 12) = 0.594; *p* = 0.456; $\eta_{p}^{2}$ = 0.047] or set [*F*(3, 36) = 0.146; *p* = 0.931; $\eta_{p}^{2}$ = 0.012] on Ek of the ball, nor was there any interaction effect [*F*(3, 36) = 0.268; *p* = 0.848; $\eta_{p}^{2}$ = 0.022] (Figure 3A). Similarly, there was no main effect of group [*F*(1, 12) = 0.031; *p* = 0.862; $\eta_{p}^{2}$ = 0.003] or set [*F*(3, 36) = 0.130; *p* = 0.942; $\eta_{p}^{2}$ = 0.011] on Et of the ball, nor was there any interaction effect [*F*(3, 36) = 0.338; *p* = 0.798; $\eta_{p}^{2}$ = 0.027] (Figure 3B).

On the other hand, a between-group main effect of Er was observed [*F*(1, 12) = 11.401; *p* = 0.006; $\eta_{p}^{2}$ = 0.487]. However, there was no main effect of the set [*F*(3, 36) = 0.754; *p* = 0.527; $\eta_{p}^{2}$ = 0.059], and no group-set interaction [*F*(3, 36) = 1.246; *p* = 0.307; $\eta_{p}^{2}$ = 0.094] was observed (Figure 3C). Similarly, a between-group main effect of %Er was observed [*F*(1, 12) = 7.766; *p* = 0.016; $\eta_{p}^{2}$ = 0.393]. However, there was no main effect of the set [*F*(3, 36) = 1.108; *p* = 0.359; $\eta_{p}^{2}$ = 0.085], and no group-set interaction [*F*(3, 36) = 1.121; *p* = 0.353; $\eta_{p}^{2}$ = 0.085] was observed (Figure 3D).

Finally, no main effect of group [*F*(1, 12) = 0.594; *p* = 0.456; $\eta_{p}^{2}$ = 0.049] or set [*F*(1.748, 20.979) = 0.475; *p* = 0.603; $\eta_{p}^{2}$ = 0.038] on distance between the target and ball landing point and no interaction effect [*F*(1.748, 20.979) = 0.852; *p* = 0.427; $\eta_{p}^{2}$ = 0.066] were observed (Figure 3F).

## Discussion

This study aimed to compare the effect of high vs. low BLa concentration in players (HIGH and LOW) (based on the initial hitting test) on the ball's kinetic energy during a hitting test and physiological responses during a simulated match-play tennis protocol. We hypothesized that players in the HIGH group would show a higher $\text{\%}E_{r}$ in the hitting test than those in the LOW group, and that running simulations would show no difference in physiological responses between groups. Supporting these hypotheses, we found that HIGH always showed higher BLa after the hitting test than LOW, $E_{k}$ was not significantly different between HIGH and LOW, $\text{\%}E_{r}$ was higher in HIGH, and physiological responses in both HIGH and LOW were associated with activity types such as running or hitting but did not depend on match progression (set progress) or differences between groups.

This study is the first to show that the rotational kinetic energy of the groundstroke is a relevant factor influencing the value of BLa in tennis. $E_{r}$ was higher in HIGH than in LOW and $E_{k}$ was comparable between groups, possibly because $E_{r}$ has much smaller magnitude than $E_{t}$. In other words, the reason for the difference in physiological responses between the two groups may have been a decrease in energy transfer efficiency between the ball and racket when applying spin to the ball, rather than an increase in the ball's rotational kinetic energy. As noted, ball-racket impact phenomena have been the subject of many studies, and the speed and spin of the ball after ball-racket impact can be estimated (13). When the kinetic energy of the ball is simulated by varying the angle of impact on the racket, mechanical energy transfer efficiency decreases as more spin is applied, as can be observationally inferred from the kinematics of the ball (14) and racket (16) when the player hits a serve. Therefore, even if the same amount of mechanical energy was applied to the ball, a player with a higher $\text{\%}E_{r}$ (HIGH) would have had to apply more mechanical energy to their racket. To obtain more mechanical energy, the glycolytic system is more rapidly utilized by mobilization of upper-body muscles required for the ball hitting action, and as a result, one would expect differences in BLa (elevation).

The accuracy and mechanical energy of the ball were not affected by the progression of the protocol (Figure 3). Therefore, it is not possible to conclude that fatigue affected the results of the hitting tests conducted in this study. Focusing on the relationship between the intensity of the hitting test and the performance of the groundstrokes, a previous study reported that the groundstroke accuracy at high intensity was reduced, but that at moderate loading the accuracy was comparable to that at rest (9). In that report, the average HR was 171 ± 7 bpm, even at moderate intensity, which would be higher than that in the hitting test in the present study. It can therefore be inferred that the load of the reported hitting test was not high enough to affect the accuracy of the groundstrokes. On the other hand, a study using a fatiguing intermittent exercise combining stroke and serve reported that stroke (accuracy: −25.6%, consistency: −15.6%) and serve (speed: −4.5%, accuracy: −11.7%) both declined over this interval (23). However, compared to the present study, which used intermittent running (approximately 1 h and 40 min), this 40-minute fatigue session included serve and groundstrokes, resulting in differences to the present study in exercise intensity, duration, and upper extremity activity. There are reports examining the decrease in serving ability in long matches, based on match statistics from actual five-set matches. While some studies reported no reduction in serve accuracy or speed between the first and fifth sets (24), some found that the average speed of first serves was higher in the first set than in the third through fifth sets (25). However, the latter study only reported an average decrease by 0.6 m/s from set 5 to set 1, and the second serve did not differ in speed between sets. Although it cannot be stated categorically, it can be thus be concluded that this study's cumulative fatigue (due to running simulation) was insufficient to affect groundstroke performance. Thus, the effects of fatigue have been the subject of disagreement in previous studies, and further investigation is needed.

In addition, no differences in groundstroke accuracy were observed between sets in this study. This may be due to the fact that the protocol did not include decision-making such as return course selection, or locomotion that occurs in an actual match, and thus presented easier conditions than under realistic conditions. Therefore, the results of this study do not allow any discussion of the effect of these factors. However, player-specific movements may be closely related to fatigue. This study showed that the type of stroke (more or less spin on the ball) affects the degree of fatigue. In a previous study that performed motion analysis before, at the midpoint, and after a 3-hour match, it was reported that the timing of maximum angular velocity was maintained before and after the match, while serving performance and joint kinetics decreased, suggesting that advanced players are able to maintain the temporal pattern of their serve even as muscle fatigue progresses (26). We therefore suggest that in future studies it is necessary to combine the analysis of movement and physiological responses.

In this study, physiological responses in both the HIGH and LOW groups were associated with activity types such as running or hitting, but did not depend on match progression (the set progress) or differ between groups. No significant differences in HR, %HRR, or VO_2_ were found between groups during the simulation protocol, suggesting that physical fitness levels were identical. However, stroke execution, which is an instantaneous movement, is an important energy-demanding factor. As Fernandez-Fernandez et al. (4) discussed, it is possible that not only the upper-body muscles required for the ball stroke but also additional muscles (e.g., biarticulate leg muscles such as biceps femoris, rectus femorus, and hip adductors during the stroke position) are required for the ball stroke. Although tennis is a combination of sprinting and hitting, a singles tennis match was found to result in higher blood glucose concentration than running (27). Therefore, differences in physiological responses between running and hitting in tennis may be caused by different metabolic demands depending on differing muscle mobilization. This is because upper-body muscle involvement is required for the ball hitting action, as indicated by previous studies. In the present study, even the basic hitting protocol with submaximal groundstroke velocity and standing position differed from running. Further study using a simulated match-play tennis protocol involving a hitting action may be warranted.

RER declined during match progression regardless of group or activity types in physiological responses. Endurance athletes can have the ability to achieve a steady state even in high-intensity interval exercise (28). Wallner et al. (29) also reported that short intermittent sprint exercise is characterized as mostly aerobically balanced exercise even if the cardiorespiratory and lactate responses oscillate intensively. Tennis is a repetitive sprint sport with medium to high aerobic and anaerobic demands (1). It is possible that tennis players in the present study, which belonged to a national level college tennis club, experienced the simulated match-play tennis protocol as mostly aerobically balanced exercise, and thus generated cardiorespiratory and lactate responses similar to the endurance athletes in the study mentioned above. This is in accordance with Ferrauti et al. (27), who showed that players of a singles tennis match demonstrated a gradual decline in RER while retaining higher glycolysis and glycogenolysis activity levels during tennis match play compared to continuous running exercise at a similar mean VO_2_ (30). However, it is possible that RER might be influenced by other factors, such as dietary fat intake, muscle glycogen content and circulating substrates (31).

## Limitations

The study is subject to several limitations. For mechanical measurements, we only measured the mechanical energy of the ball and did not take the energetics of the player's body motion into account. In addition, because individual subjects were not measured when applying different ratios of spin to speed, there may have been player-specific differences in form. In future studies, it will be necessary to have the same subjects hit groundstrokes with different ratios of spin and speed and to compare the mechanical work. In the physiological part of the study, players performed running and hitting groundstrokes fed by a ball machine during the warm-up period. The effort of running in the warm-up was not standardized, while hitting groundstrokes was set up similar to the hitting test in order to ensure equal effort. Because maximum HR was not measured by a graded exercise test before the hitting test, we employed the commonly used equation of HRmax = 220- age for prediction. This equation is frequently used in prescribing exercise intensity, but is acknowledged to be quite variable in its accuracy, with estimates having a standard deviation of 10–12 bpm (32), and the validity of alternative formulas is under discussion (33). In addition, since there exist maximum HR equations that differentiate between genders (34), further research should employ these more specific equations.

## Conclusion

In this study, we hypothesized that the amount of ball spin on groundstrokes would be related to blood lactate concentration. We tested this hypothesis by combining a match simulation protocol and hitting tests. In support of this hypothesis, despite no difference in $E_{k}$ between groups, $\text{\%}E_{r}$ was higher in the high BLa group than in the low BLa group. In addition, the progression of the simulation protocol did not affect the result of the hitting test, and there were no significant changes in physiological responses. In other words, simple hitting ability, which does not include decisions, is not affected by the progression of the game, but may be more influenced by the activity intensity of the previous match point. The effect of fatigue on hitting ability has been controversial in previous studies, but in any case, a match simulation protocol that does not include a hitting task may not be able to accurately evaluate comparative game progression between players who do or do not apply heavy spin to the ball on a groundstroke.

Our results suggest that physiological load differs depending on the type of groundstroke (high or low spin rates). Therefore, players should plan their fitness training according to their groundstroke type. In addition, as discussed with regard to the importance of pacing on the serve (25), it seems essential to adjust the spin rate of groundstrokes according to fatigue during the match, qualified by game strategy and other factors.

## Data Availability

The raw data supporting the conclusions of this article will be made available by the authors, without undue reservation.
